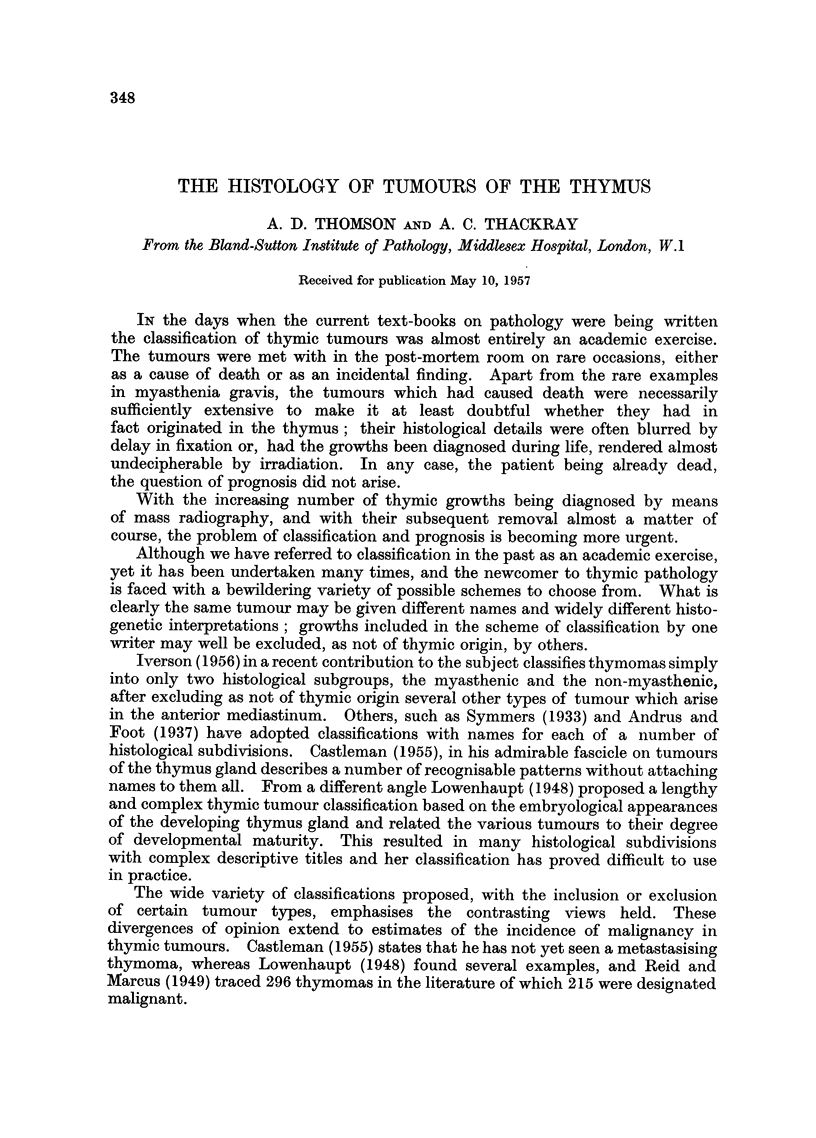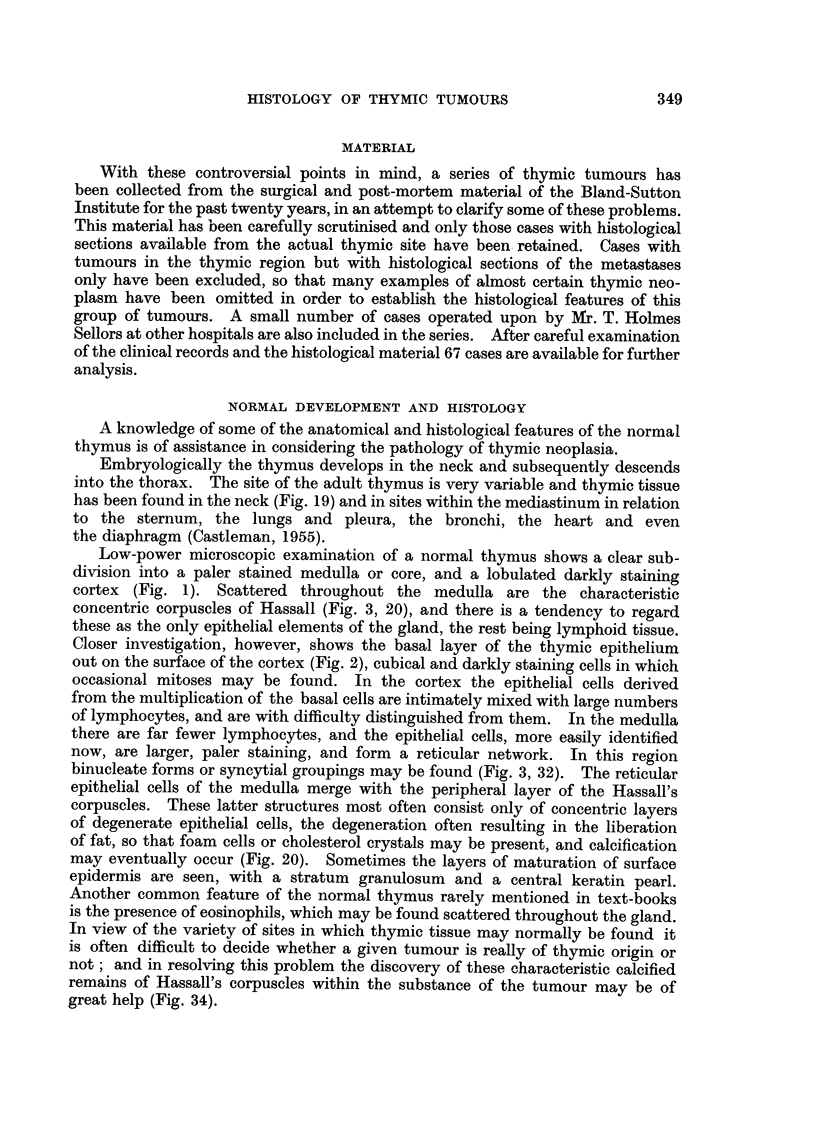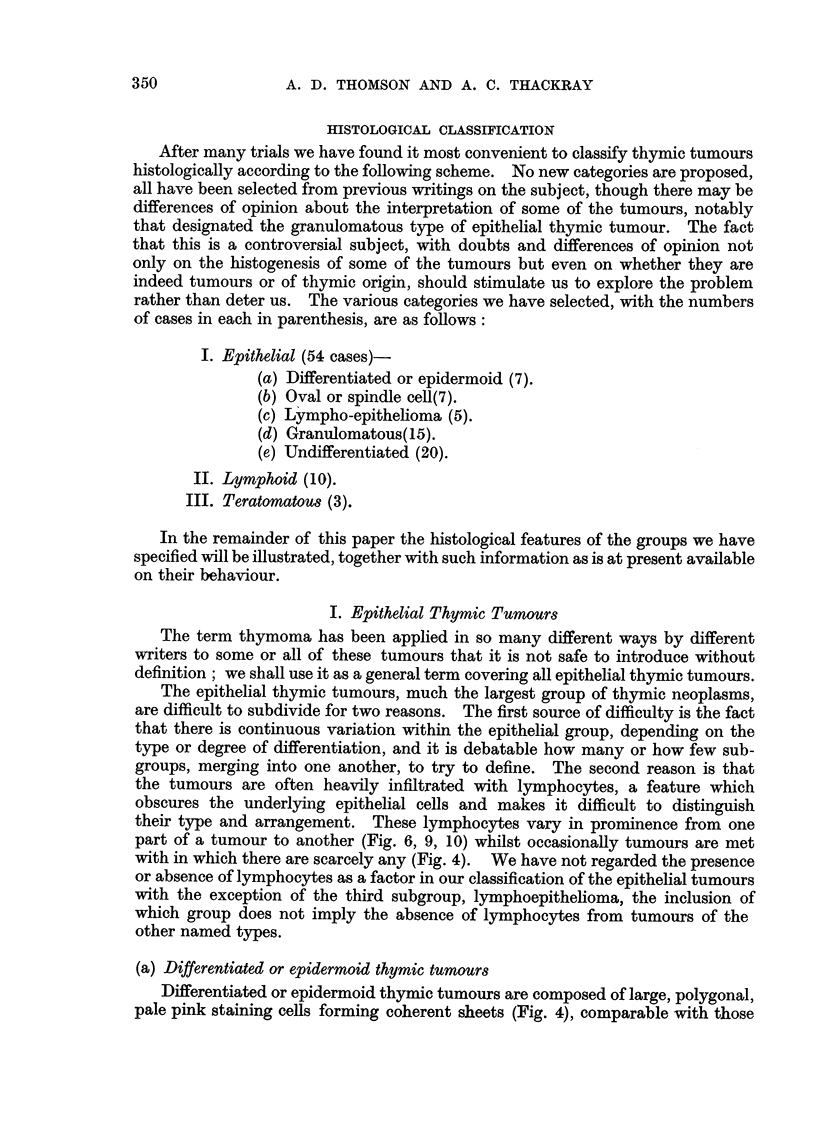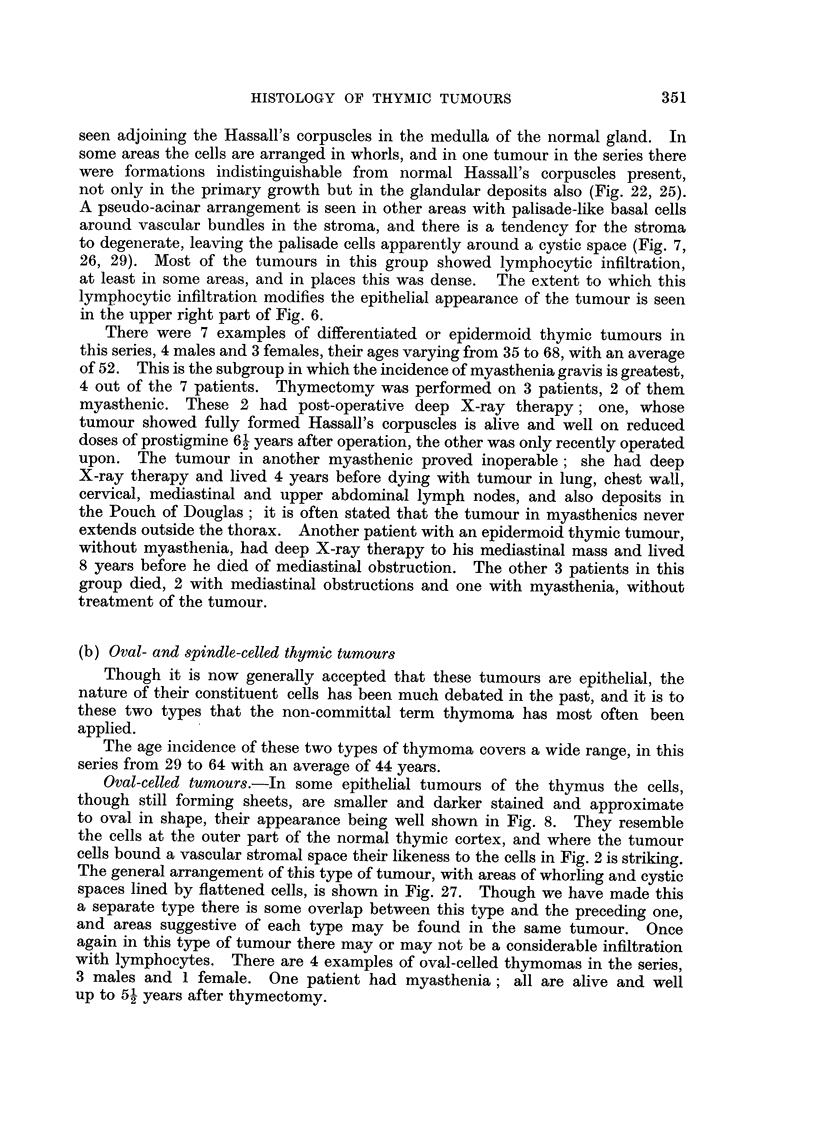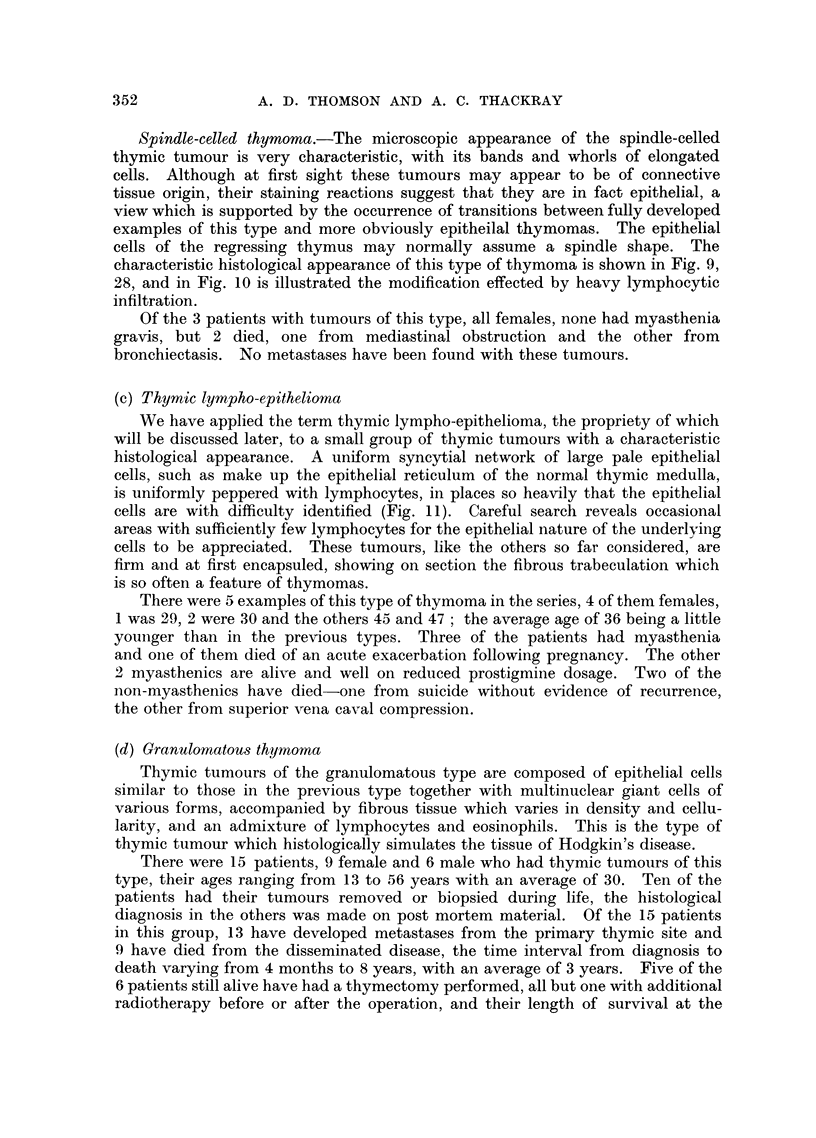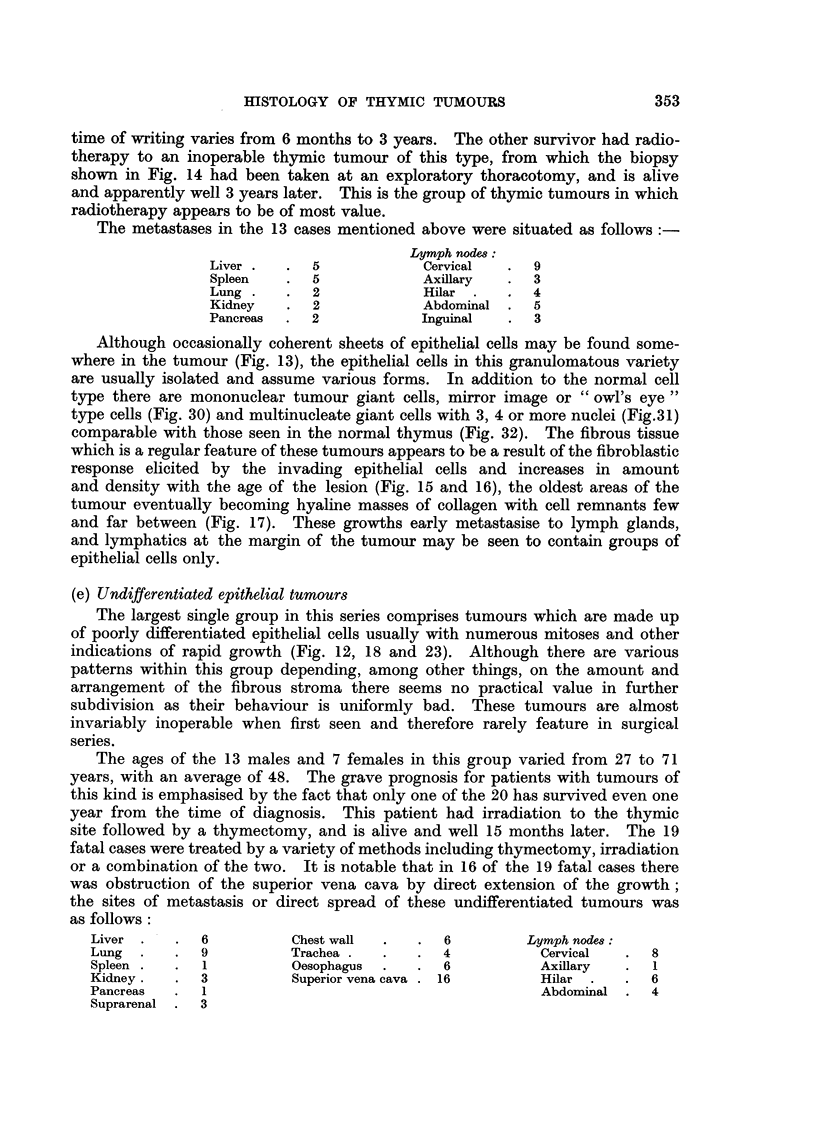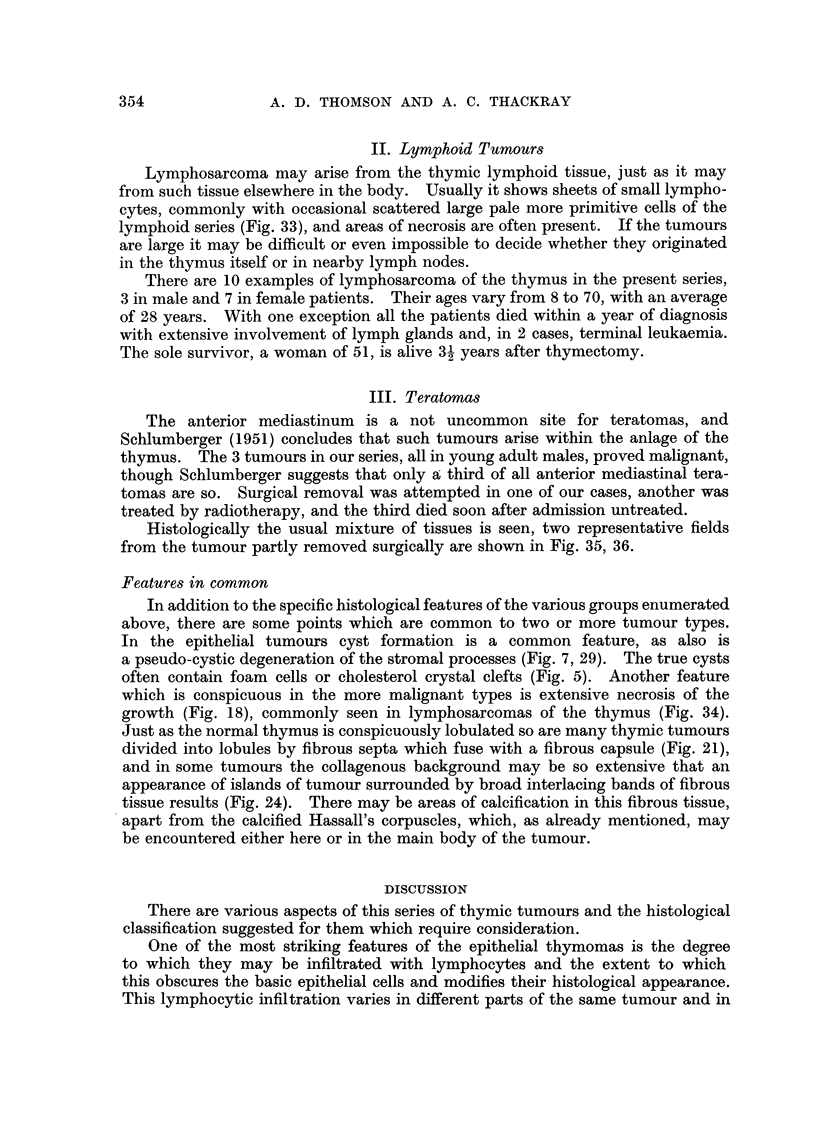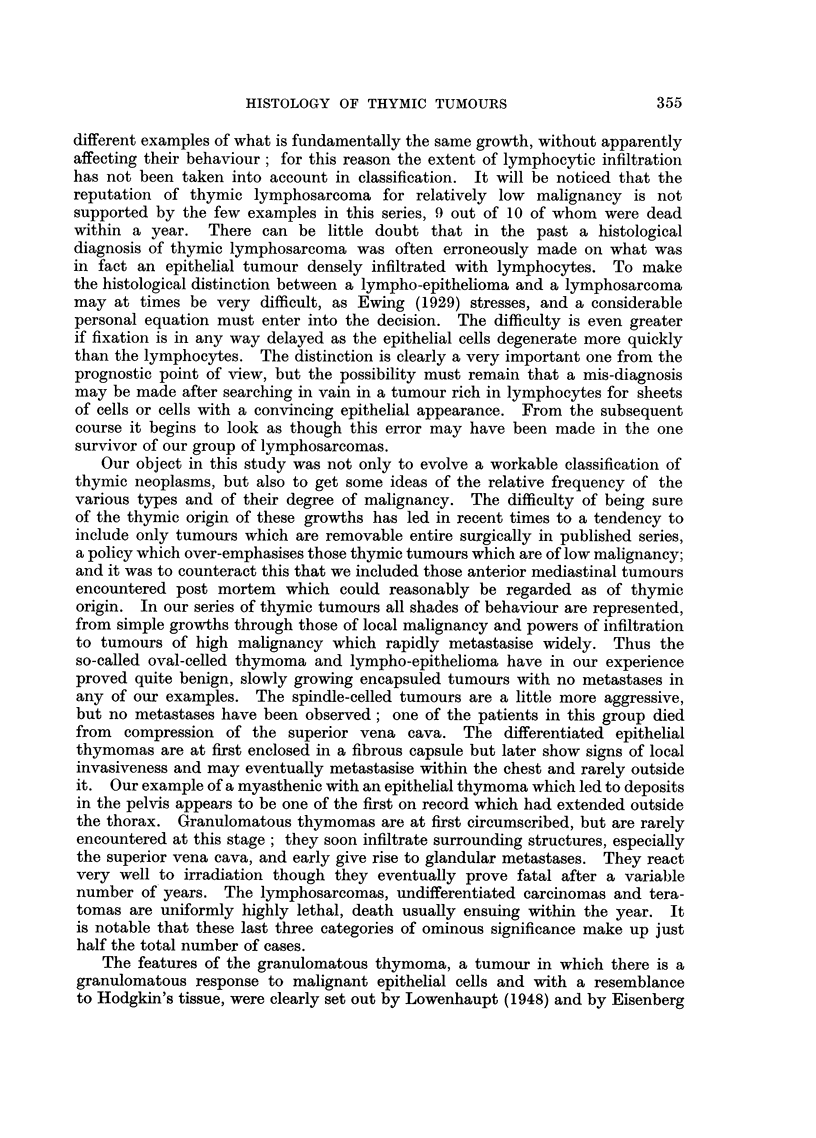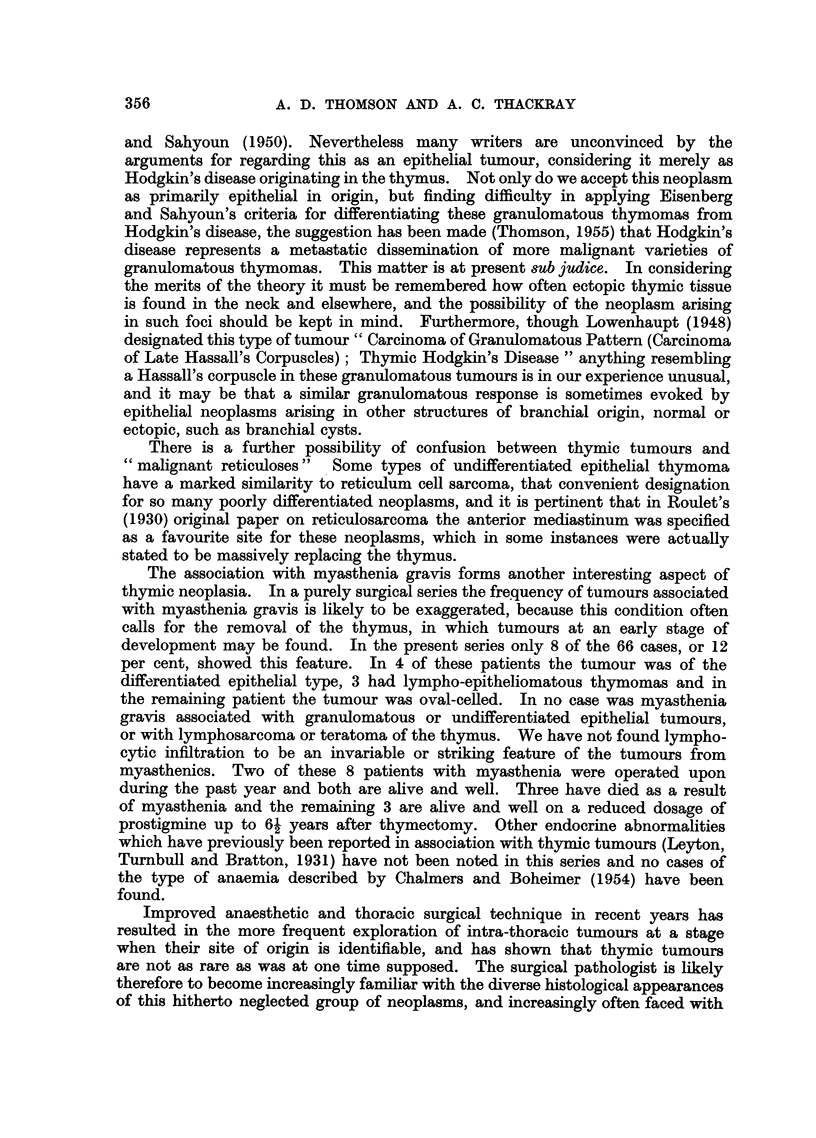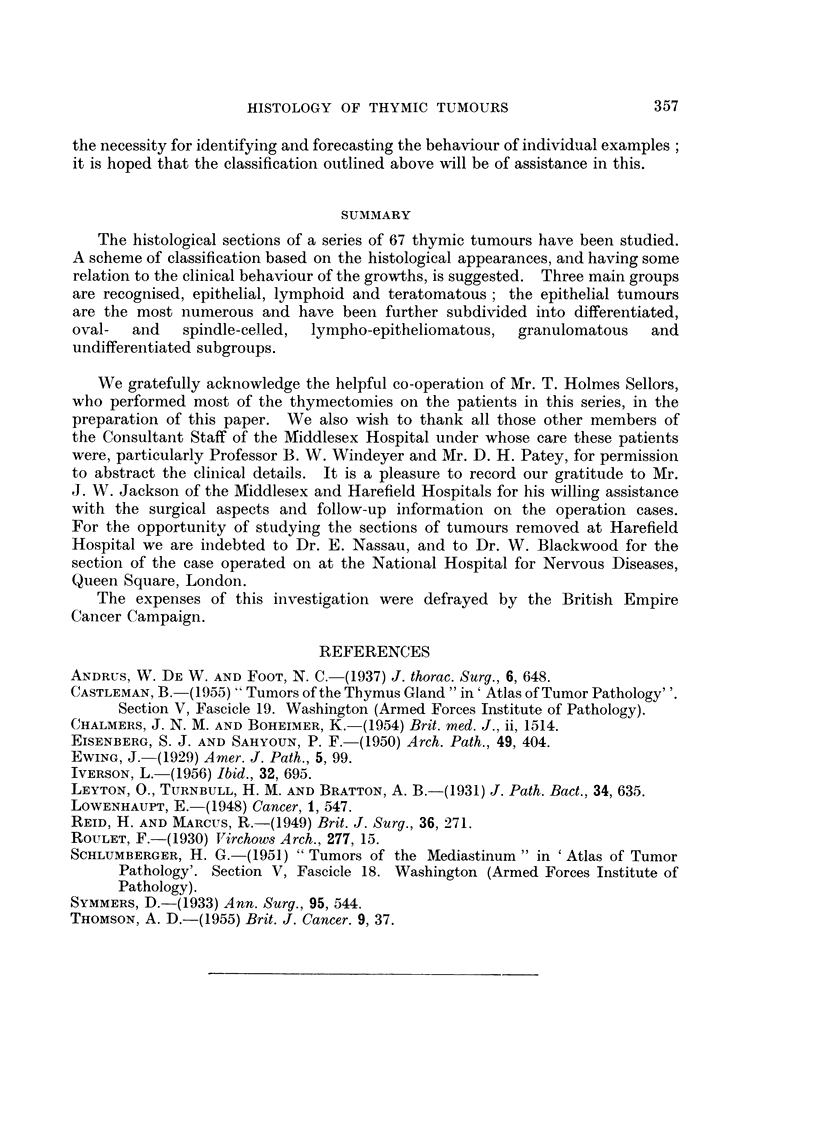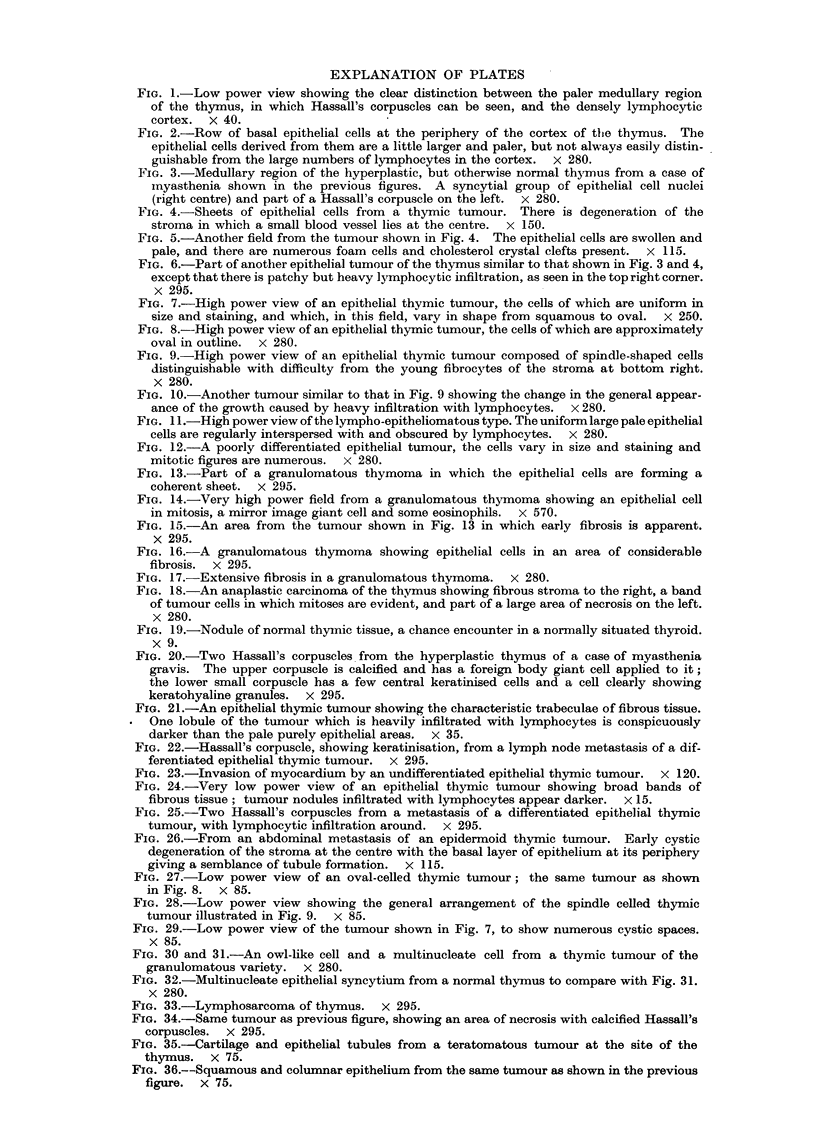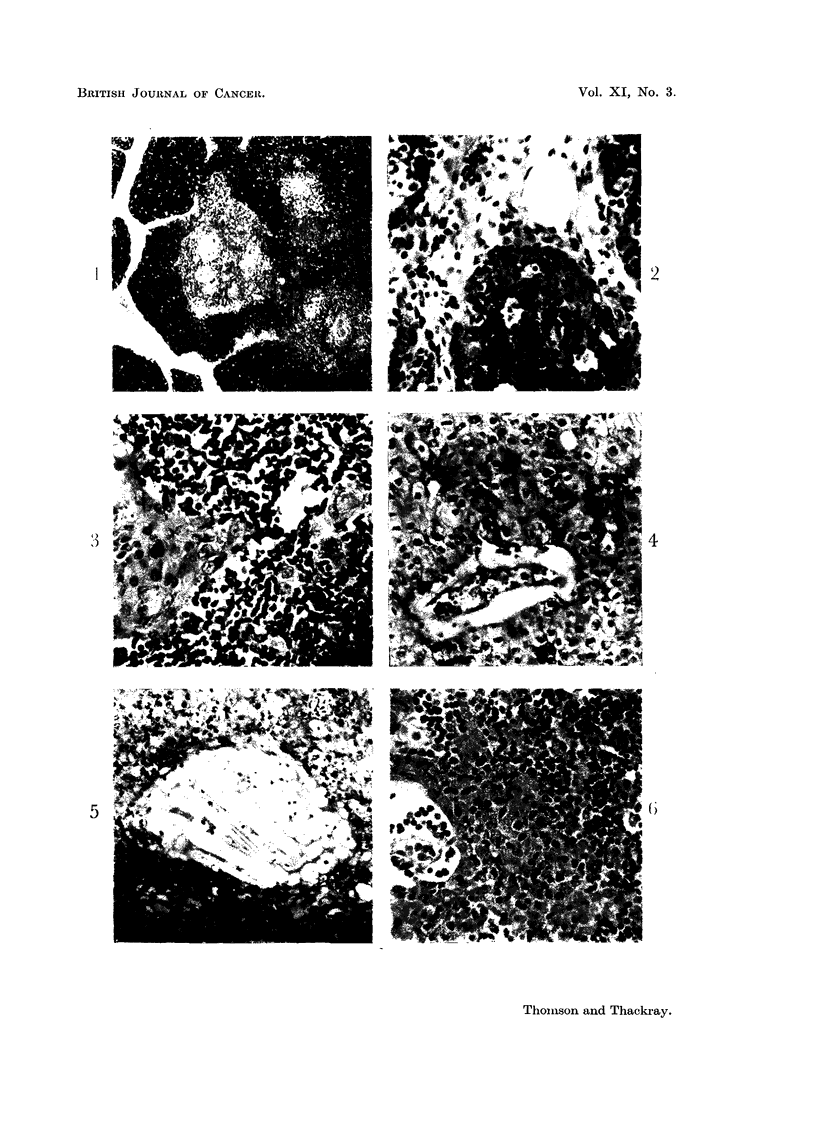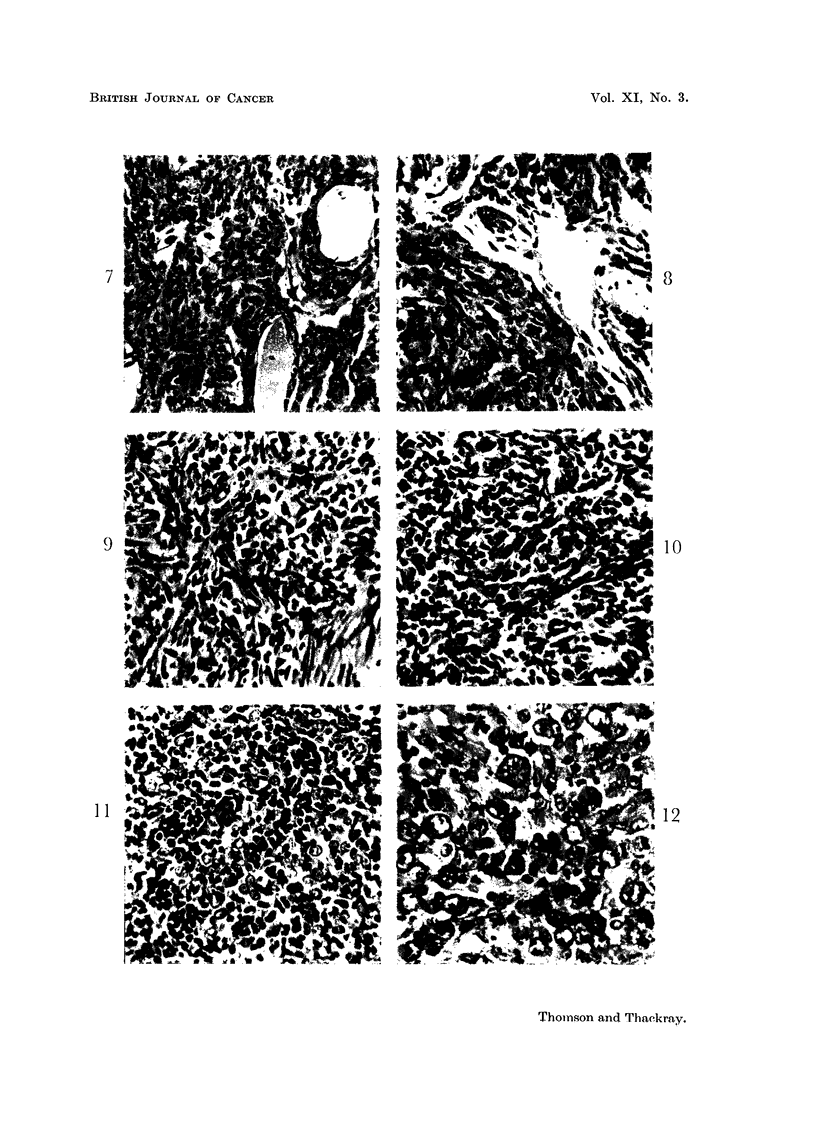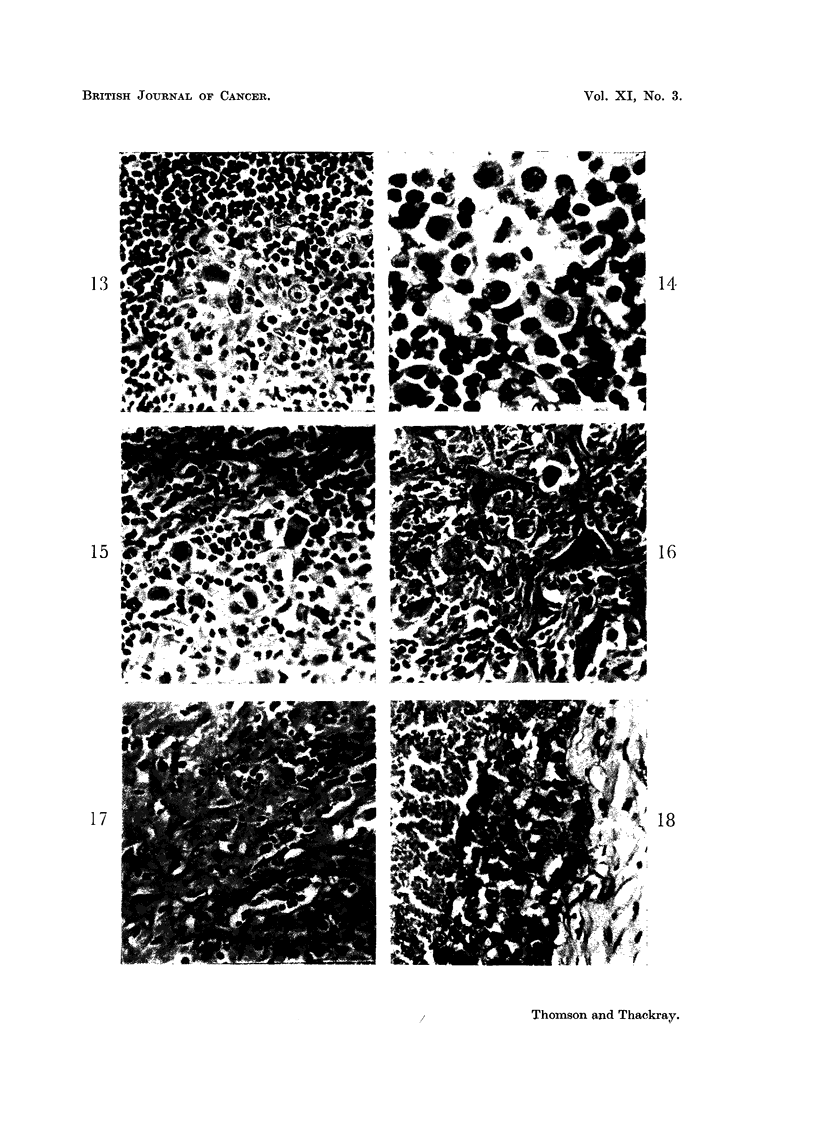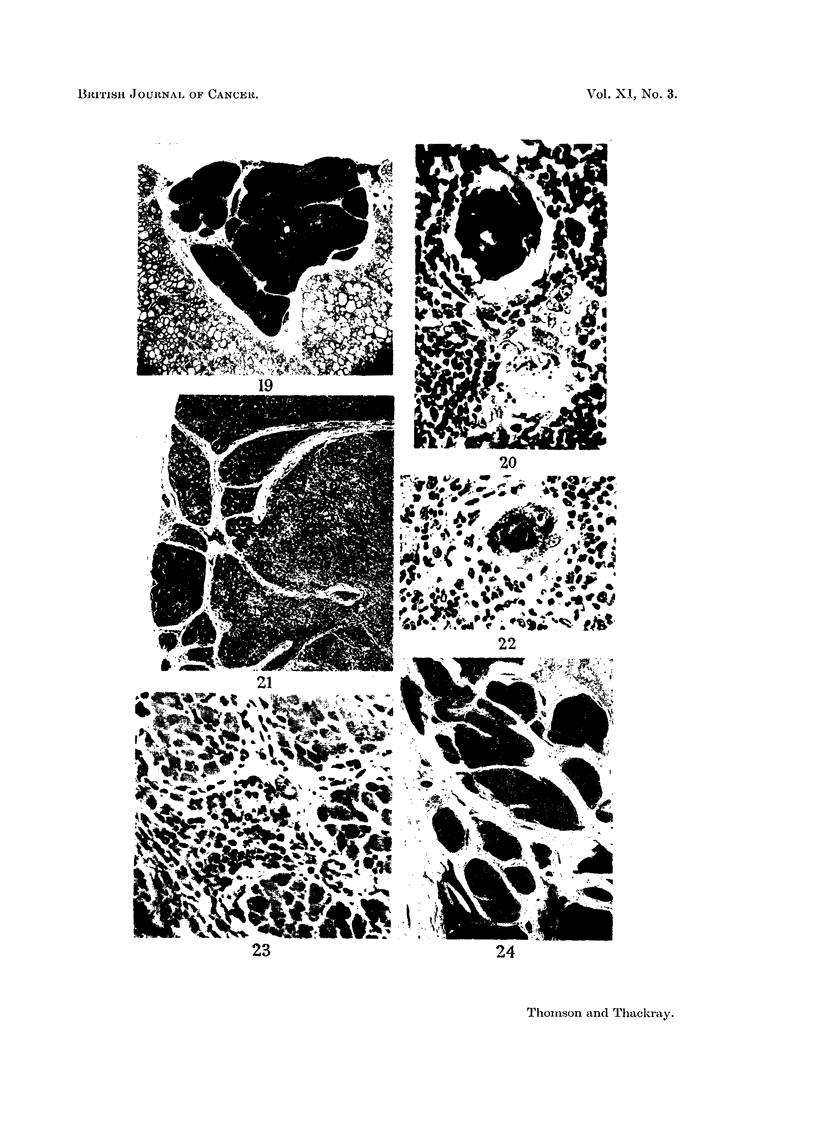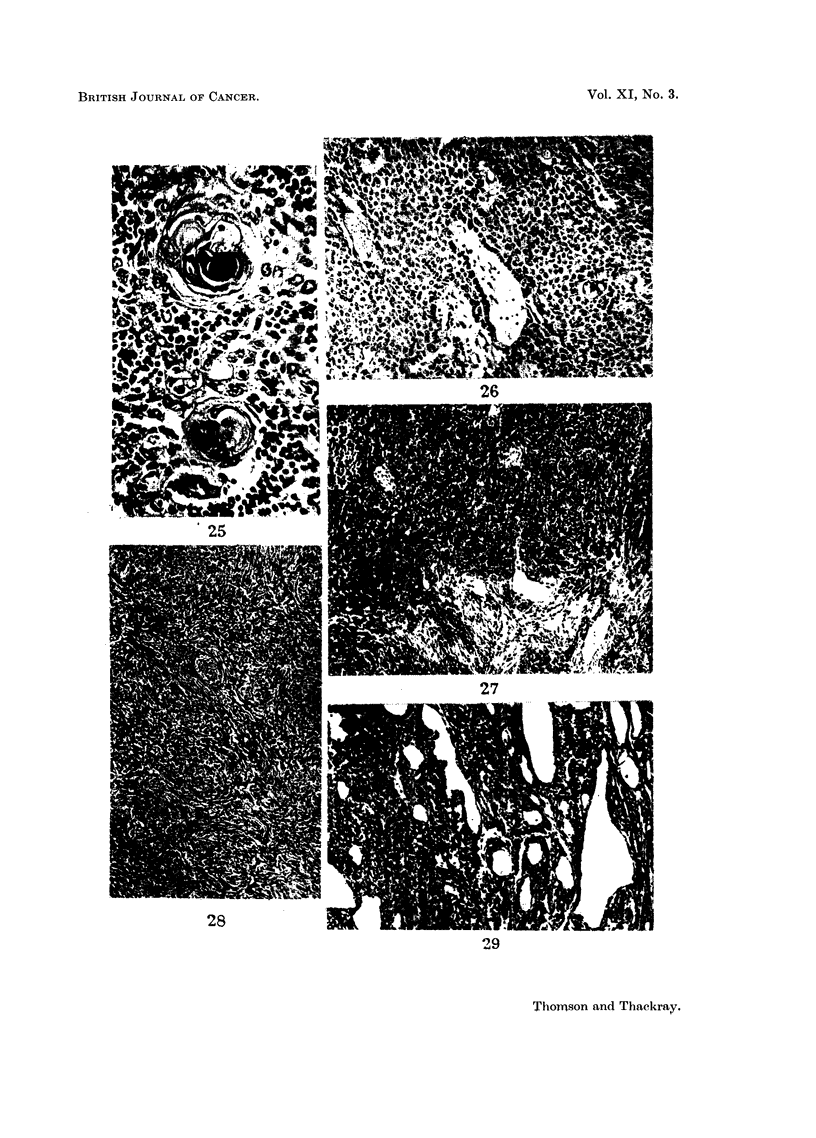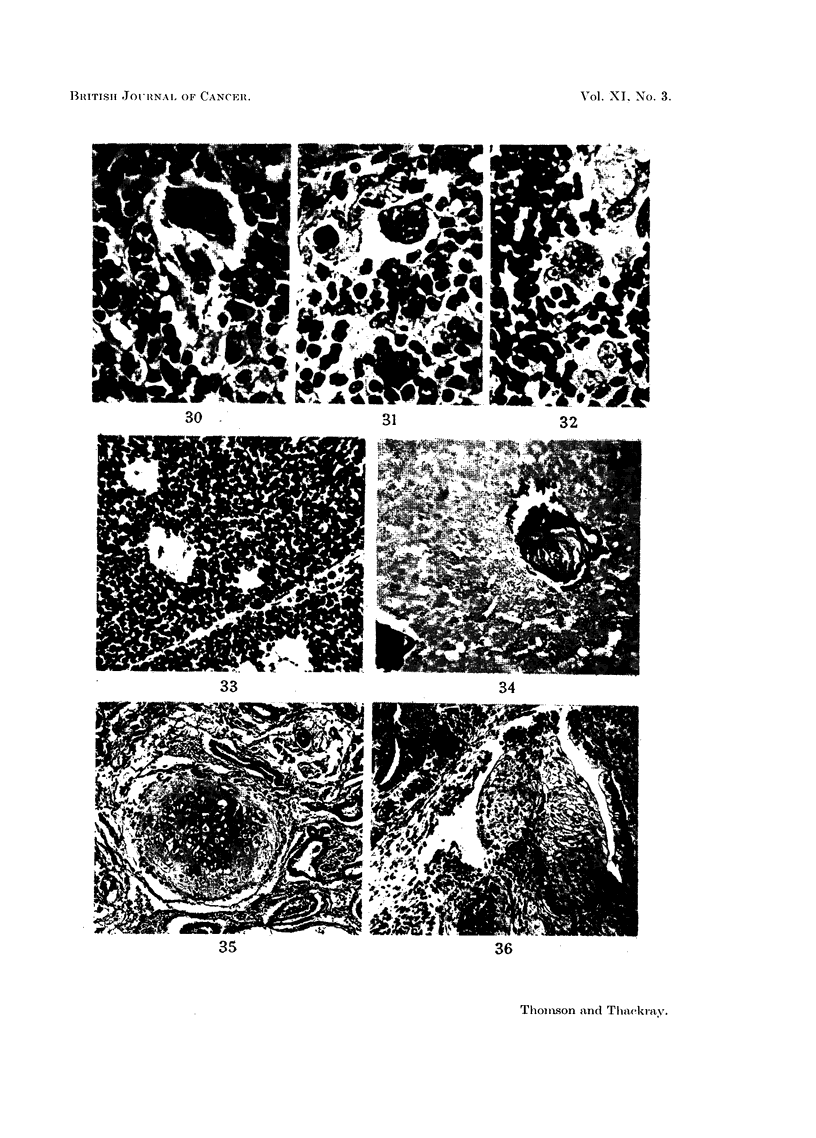# The Histology of Tumours of the Thymus

**DOI:** 10.1038/bjc.1957.42

**Published:** 1957-09

**Authors:** A. D. Thomson, A. C. Thackray

## Abstract

**Images:**


### THE HISTOLOGY OF TUMOURS OF THE THYMUS

A. D. THOMSON AND A. C. THACKRAY
*From the Bland-Sutton Institute of Pathology, Middlesex Hospital, London, W.1*

Received for publication May 10, 1957

IN the days when the current text-books on pathology were being written the classification of thymic tumours was almost entirely an academic exercise. The tumours were met with in the post-mortem room on rare occasions, either as a cause of death or as an incidental finding. Apart from the rare examples in myasthenia gravis, the tumours which had caused death were necessarily sufficiently extensive to make it at least doubtful whether they had in fact originated in the thymus ; their histological details were often blurred by delay in fixation or, had the growths been diagnosed during life, rendered almost undecipherable by irradiation. In any case, the patient being already dead, the question of prognosis did not arise.

With the increasing number of thymic growths being diagnosed by means of mass radiography, and with their subsequent removal almost a matter of course, the problem of classification and prognosis is becoming more urgent.

Although we have referred to classification in the past as an academic exercise, yet it has been undertaken many times, and the newcomer to thymic pathology is faced with a bewildering variety of possible schemes to choose from. What is clearly the same tumour may be given different names and widely different histogenetic interpretations ; growths included in the scheme of classification by one writer may well be excluded, as not of thymic origin, by others.

Iverson (1956) in a recent contribution to the subject classifies thymomas simply into only two histological subgroups, the myasthenic and the non-myasthenic, after excluding as not of thymic origin several other types of tumour which arise in the anterior mediastinum. Others, such as Symmers (1933) and Andrus and Foot (1937) have adopted classifications with names for each of a number of histological subdivisions. Castleman (1955), in his admirable fascicle on tumours of the thymus gland describes a number of recognisable patterns without attaching names to them all. From a different angle Lowenhaupt (1948) proposed a lengthy and complex thymic tumour classification based on the embryological appearances of the developing thymus gland and related the various tumours to their degree of developmental maturity. This resulted in many histological subdivisions with complex descriptive titles and her classification has proved difficult to use in practice.

The wide variety of classifications proposed, with the inclusion or exclusion of certain tumour types, emphasises the contrasting views held. These divergences of opinion extend to estimates of the incidence of malignancy in thymic tumours. Castleman (1955) states that he has not yet seen a metastasising thymoma, whereas Lowenhaupt (1948) found several examples, and Reid and Marcus (1949) traced 296 thymomas in the literature of which 215 were designated malignant.

### MATERIAL

With these controversial points in mind, a series of thymic tumours has been collected from the surgical and post-mortem material of the Bland-Sutton Institute for the past twenty years, in an attempt to clarify some of these problems. This material has been carefully scrutinised and only those cases with histological sections available from the actual thymic site have been retained. Cases with tumours in the thymic region but with histological sections of the metastases only have been excluded, so that many examples of almost certain thymic neoplasm have been omitted in order to establish the histological features of this group of tumours. A small number of cases operated upon by Mr. T. Holmes Sellors at other hospitals are also included in the series. After careful examination of the clinical records and the histological material 67 cases are available for further analysis.

### NORMAL DEVELOPMENT AND HISTOLOGY

A knowledge of some of the anatomical and histological features of the normal thymus is of assistance in considering the pathology of thymic neoplasia.

Embryologically the thymus develops in the neck and subsequently descends into the thorax. The site of the adult thymus is very variable and thymic tissue has been found in the neck (Fig. 19) and in sites within the mediastinum in relation to the sternum, the lungs and pleura, the bronchi, the heart and even the diaphragm (Castleman, 1955).

Low-power microscopic examination of a normal thymus shows a clear subdivision into a paler stained medulla or core, and a lobulated darkly staining cortex (Fig. 1). Scattered throughout the medulla are the characteristic concentric corpuscles of Hassall (Fig. 3, 20), and there is a tendency to regard these as the only epithelial elements of the gland, the rest being lymphoid tissue. Closer investigation, however, shows the basal layer of the thymic epithelium out on the surface of the cortex (Fig. 2), cubical and darkly staining cells in which occasional mitoses may be found. In the cortex the epithelial cells derived from the multiplication of the basal cells are intimately mixed with large numbers of lymphocytes, and are with difficulty distinguished from them. In the medulla there are far fewer lymphocytes, and the epithelial cells, more easily identified now, are larger, paler staining, and form a reticular network. In this region binucleate forms or syncytial groupings may be found (Fig. 3, 32). The reticular epithelial cells of the medulla merge with the peripheral layer of the Hassall's corpuscles. These latter structures most often consist only of concentric layers of degenerate epithelial cells, the degeneration often resulting in the liberation of fat, so that foam cells or cholesterol crystals may be present, and calcification may eventually occur (Fig. 20). Sometimes the layers of maturation of surface epidermis are seen, with a stratum granulosum and a central keratin pearl. Another common feature of the normal thymus rarely mentioned in text-books is the presence of eosinophils, which may be found scattered throughout the gland. In view of the variety of sites in which thymic tissue may normally be found it is often difficult to decide whether a given tumour is really of thymic origin or not ; and in resolving this problem the discovery of these characteristic calcified remains of Hassall's corpuscles within the substance of the tumour may be of great help (Fig. 34).

### HISTOLOGICAL CLASSIFICATION

After many trials we have found it most convenient to classify thymic tumours histologically according to the following scheme. No new categories are proposed, all have been selected from previous writings on the subject, though there may be differences of opinion about the interpretation of some of the tumours, notably that designated the granulomatous type of epithelial thymic tumour. The fact that this is a controversial subject, with doubts and differences of opinion not only on the histogenesis of some of the tumours but even on whether they are indeed tumours or of thymic origin, should stimulate us to explore the problem rather than deter us. The various categories we have selected, with the numbers of cases in each in parenthesis, are as follows :

I. *Epithelial* (54 cases)—
- (*a*) Differentiated or epidermoid (7).
- (*b*) Oval or spindle cell(7).
- (*c*) Lympho-epithelioma (5).
- (*d*) Granulomatous(15).
- (*e*) Undifferentiated (20).

II. *Lymphoid* (10).

III. *Teratomatous* (3).

In the remainder of this paper the histological features of the groups we have specified will be illustrated, together with such information as is at present available on their behaviour.

### I. *Epithelial Thymic Tumours*

The term thymoma has been applied in so many different ways by different writers to some or all of these tumours that it is not safe to introduce without definition ; we shall use it as a general term covering all epithelial thymic tumours.

The epithelial thymic tumours, much the largest group of thymic neoplasms, are difficult to subdivide for two reasons. The first source of difficulty is the fact that there is continuous variation within the epithelial group, depending on the type or degree of differentiation, and it is debatable how many or how few sub-groups, merging into one another, to try to define. The second reason is that the tumours are often heavily infiltrated with lymphocytes, a feature which obscures the underlying epithelial cells and makes it difficult to distinguish their type and arrangement. These lymphocytes vary in prominence from one part of a tumour to another (Fig. 6, 9, 10) whilst occasionally tumours are met with in which there are scarcely any (Fig. 4). We have not regarded the presence or absence of lymphocytes as a factor in our classification of the epithelial tumours with the exception of the third subgroup, lymphoepithelioma, the inclusion of which group does not imply the absence of lymphocytes from tumours of the other named types.

#### (a) *Differentiated or epidermoid thymic tumours*

Differentiated or epidermoid thymic tumours are composed of large, polygonal, pale pink staining cells forming coherent sheets (Fig. 4), comparable with those seen adjoining the Hassall's corpuscles in the medulla of the normal gland. In some areas the cells are arranged in whorls, and in one tumour in the series there were formations indistinguishable from normal Hassall's corpuscles present, not only in the primary growth but in the glandular deposits also (Fig. 22, 25). A pseudo-acinar arrangement is seen in other areas with palisade-like basal cells around vascular bundles in the stroma, and there is a tendency for the stroma to degenerate, leaving the palisade cells apparently around a cystic space (Fig. 7, 26, 29). Most of the tumours in this group showed lymphocytic infiltration, at least in some areas, and in places this was dense. The extent to which this lymphocytic infiltration modifies the epithelial appearance of the tumour is seen in the upper right part of Fig. 6.

There were 7 examples of differentiated or epidermoid thymic tumours in this series, 4 males and 3 females, their ages varying from 35 to 68, with an average of 52. This is the subgroup in which the incidence of myasthenia gravis is greatest, 4 out of the 7 patients. Thymectomy was performed on 3 patients, 2 of them myasthenic. These 2 had post-operative deep X-ray therapy; one, whose tumour showed fully formed Hassall's corpuscles is alive and well on reduced doses of prostigmine 6½ years after operation, the other was only recently operated upon. The tumour in another myasthenic proved inoperable; she had deep X-ray therapy and lived 4 years before dying with tumour in lung, chest wall, cervical, mediastinal and upper abdominal lymph nodes, and also deposits in the Pouch of Douglas; it is often stated that the tumour in myasthenics never extends outside the thorax. Another patient with an epidermoid thymic tumour, without myasthenia, had deep X-ray therapy to his mediastinal mass and lived 8 years before he died of mediastinal obstruction. The other 3 patients in this group died, 2 with mediastinal obstructions and one with myasthenia, without treatment of the tumour.

(b) *Oval- and spindle-celled thymic tumours*

Though it is now generally accepted that these tumours are epithelial, the nature of their constituent cells has been much debated in the past, and it is to these two types that the non-committal term thymoma has most often been applied.

The age incidence of these two types of thymoma covers a wide range, in this series from 29 to 64 with an average of 44 years.

*Oval-celled tumours.*—In some epithelial tumours of the thymus the cells, though still forming sheets, are smaller and darker stained and approximate to oval in shape, their appearance being well shown in Fig. 8. They resemble the cells at the outer part of the normal thymic cortex, and where the tumour cells bound a vascular stromal space their likeness to the cells in Fig. 2 is striking. The general arrangement of this type of tumour, with areas of whorling and cystic spaces lined by flattened cells, is shown in Fig. 27. Though we have made this a separate type there is some overlap between this type and the preceding one, and areas suggestive of each type may be found in the same tumour. Once again in this type of tumour there may or may not be a considerable infiltration with lymphocytes. There are 4 examples of oval-celled thymomas in the series, 3 males and 1 female. One patient had myasthenia; all are alive and well up to 5½ years after thymectomy.

*Spindle-celled thymoma.*—The microscopic appearance of the spindle-celled thymic tumour is very characteristic, with its bands and whorls of elongated cells. Although at first sight these tumours may appear to be of connective tissue origin, their staining reactions suggest that they are in fact epithelial, a view which is supported by the occurrence of transitions between fully developed examples of this type and more obviously epitheilal thymomas. The epithelial cells of the regressing thymus may normally assume a spindle shape. The characteristic histological appearance of this type of thymoma is shown in Fig. 9, 28, and in Fig. 10 is illustrated the modification effected by heavy lymphocytic infiltration.

Of the 3 patients with tumours of this type, all females, none had myasthenia gravis, but 2 died, one from mediastinal obstruction and the other from bronchiectasis. No metastases have been found with these tumours.

(c) *Thymic lympho-epithelioma*

We have applied the term thymic lympho-epithelioma, the propriety of which will be discussed later, to a small group of thymic tumours with a characteristic histological appearance. A uniform syncytial network of large pale epithelial cells, such as make up the epithelial reticulum of the normal thymic medulla, is uniformly peppered with lymphocytes, in places so heavily that the epithelial cells are with difficulty identified (Fig. 11). Careful search reveals occasional areas with sufficiently few lymphocytes for the epithelial nature of the underlying cells to be appreciated. These tumours, like the others so far considered, are firm and at first encapsuled, showing on section the fibrous trabeculation which is so often a feature of thymomas.

There were 5 examples of this type of thymoma in the series, 4 of them females, 1 was 29, 2 were 30 and the others 45 and 47 ; the average age of 36 being a little younger than in the previous types. Three of the patients had myasthenia and one of them died of an acute exacerbation following pregnancy. The other 2 myasthenics are alive and well on reduced prostigmine dosage. Two of the non-myasthenics have died—one from suicide without evidence of recurrence, the other from superior vena caval compression.

(*d*) *Granulomatous thymoma*

Thymic tumours of the granulomatous type are composed of epithelial cells similar to those in the previous type together with multinuclear giant cells of various forms, accompanied by fibrous tissue which varies in density and cellularity, and an admixture of lymphocytes and eosinophils. This is the type of thymic tumour which histologically simulates the tissue of Hodgkin's disease.

There were 15 patients, 9 female and 6 male who had thymic tumours of this type, their ages ranging from 13 to 56 years with an average of 30. Ten of the patients had their tumours removed or biopsied during life, the histological diagnosis in the others was made on post mortem material. Of the 15 patients in this group, 13 have developed metastases from the primary thymic site and 9 have died from the disseminated disease, the time interval from diagnosis to death varying from 4 months to 8 years, with an average of 3 years. Five of the 6 patients still alive have had a thymectomy performed, all but one with additional radiotherapy before or after the operation, and their length of survival at the time of writing varies from 6 months to 3 years. The other survivor had radiotherapy to an inoperable thymic tumour of this type, from which the biopsy shown in Fig. 14 had been taken at an exploratory thoracotomy, and is alive and apparently well 3 years later. This is the group of thymic tumours in which radiotherapy appears to be of most value.

The metastases in the 13 cases mentioned above were situated as follows :—

| | | | |
|---|---|---|---|
| | | *Lymph nodes :* | |
| Liver | 5 | Cervical | 9 |
| Spleen | 5 | Axillary | 3 |
| Lung | 2 | Hilar | 4 |
| Kidney | 2 | Abdominal | 5 |
| Pancreas | 2 | Inguinal | 3 |

Although occasionally coherent sheets of epithelial cells may be found somewhere in the tumour (Fig. 13), the epithelial cells in this granulomatous variety are usually isolated and assume various forms. In addition to the normal cell type there are mononuclear tumour giant cells, mirror image or " owl's eye " type cells (Fig. 30) and multinucleate giant cells with 3, 4 or more nuclei (Fig.31) comparable with those seen in the normal thymus (Fig. 32). The fibrous tissue which is a regular feature of these tumours appears to be a result of the fibroblastic response elicited by the invading epithelial cells and increases in amount and density with the age of the lesion (Fig. 15 and 16), the oldest areas of the tumour eventually becoming hyaline masses of collagen with cell remnants few and far between (Fig. 17). These growths early metastasise to lymph glands, and lymphatics at the margin of the tumour may be seen to contain groups of epithelial cells only.

(e) *Undifferentiated epithelial tumours*

The largest single group in this series comprises tumours which are made up of poorly differentiated epithelial cells usually with numerous mitoses and other indications of rapid growth (Fig. 12, 18 and 23). Although there are various patterns within this group depending, among other things, on the amount and arrangement of the fibrous stroma there seems no practical value in further subdivision as their behaviour is uniformly bad. These tumours are almost invariably inoperable when first seen and therefore rarely feature in surgical series.

The ages of the 13 males and 7 females in this group varied from 27 to 71 years, with an average of 48. The grave prognosis for patients with tumours of this kind is emphasised by the fact that only one of the 20 has survived even one year from the time of diagnosis. This patient had irradiation to the thymic site followed by a thymectomy, and is alive and well 15 months later. The 19 fatal cases were treated by a variety of methods including thymectomy, irradiation or a combination of the two. It is notable that in 16 of the 19 fatal cases there was obstruction of the superior vena cava by direct extension of the growth ; the sites of metastasis or direct spread of these undifferentiated tumours was as follows :

| | | | | | |
|---|---|---|---|---|---|
| Liver | 6 | Chest wall | 6 | *Lymph nodes :* | |
| Lung | 9 | Trachea | 4 | Cervical | 8 |
| Spleen | 1 | Oesophagus | 6 | Axillary | 1 |
| Kidney | 3 | Superior vena cava | 16 | Hilar | 6 |
| Pancreas | 1 | | | Abdominal | 4 |
| Suprarenal | 3 | | | | |

### II. *Lymphoid Tumours*

Lymphosarcoma may arise from the thymic lymphoid tissue, just as it may from such tissue elsewhere in the body. Usually it shows sheets of small lymphocytes, commonly with occasional scattered large pale more primitive cells of the lymphoid series (Fig. 33), and areas of necrosis are often present. If the tumours are large it may be difficult or even impossible to decide whether they originated in the thymus itself or in nearby lymph nodes.

There are 10 examples of lymphosarcoma of the thymus in the present series, 3 in male and 7 in female patients. Their ages vary from 8 to 70, with an average of 28 years. With one exception all the patients died within a year of diagnosis with extensive involvement of lymph glands and, in 2 cases, terminal leukaemia. The sole survivor, a woman of 51, is alive $3\frac{1}{2}$ years after thymectomy.

### III. *Teratomas*

The anterior mediastinum is a not uncommon site for teratomas, and Schlumberger (1951) concludes that such tumours arise within the anlage of the thymus. The 3 tumours in our series, all in young adult males, proved malignant, though Schlumberger suggests that only a third of all anterior mediastinal teratomas are so. Surgical removal was attempted in one of our cases, another was treated by radiotherapy, and the third died soon after admission untreated.

Histologically the usual mixture of tissues is seen, two representative fields from the tumour partly removed surgically are shown in Fig. 35, 36.

*Features in common*

In addition to the specific histological features of the various groups enumerated above, there are some points which are common to two or more tumour types. In the epithelial tumours cyst formation is a common feature, as also is a pseudo-cystic degeneration of the stromal processes (Fig. 7, 29). The true cysts often contain foam cells or cholesterol crystal clefts (Fig. 5). Another feature which is conspicuous in the more malignant types is extensive necrosis of the growth (Fig. 18), commonly seen in lymphosarcomas of the thymus (Fig. 34). Just as the normal thymus is conspicuously lobulated so are many thymic tumours divided into lobules by fibrous septa which fuse with a fibrous capsule (Fig. 21), and in some tumours the collagenous background may be so extensive that an appearance of islands of tumour surrounded by broad interlacing bands of fibrous tissue results (Fig. 24). There may be areas of calcification in this fibrous tissue, apart from the calcified Hassall's corpuscles, which, as already mentioned, may be encountered either here or in the main body of the tumour.

### DISCUSSION

There are various aspects of this series of thymic tumours and the histological classification suggested for them which require consideration.

One of the most striking features of the epithelial thymomas is the degree to which they may be infiltrated with lymphocytes and the extent to which this obscures the basic epithelial cells and modifies their histological appearance. This lymphocytic infiltration varies in different parts of the same tumour and in different examples of what is fundamentally the same growth, without apparently affecting their behaviour ; for this reason the extent of lymphocytic infiltration has not been taken into account in classification. It will be noticed that the reputation of thymic lymphosarcoma for relatively low malignancy is not supported by the few examples in this series, 9 out of 10 of whom were dead within a year. There can be little doubt that in the past a histological diagnosis of thymic lymphosarcoma was often erroneously made on what was in fact an epithelial tumour densely infiltrated with lymphocytes. To make the histological distinction between a lympho-epithelioma and a lymphosarcoma may at times be very difficult, as Ewing (1929) stresses, and a considerable personal equation must enter into the decision. The difficulty is even greater if fixation is in any way delayed as the epithelial cells degenerate more quickly than the lymphocytes. The distinction is clearly a very important one from the prognostic point of view, but the possibility must remain that a mis-diagnosis may be made after searching in vain in a tumour rich in lymphocytes for sheets of cells or cells with a convincing epithelial appearance. From the subsequent course it begins to look as though this error may have been made in the one survivor of our group of lymphosarcomas.

Our object in this study was not only to evolve a workable classification of thymic neoplasms, but also to get some ideas of the relative frequency of the various types and of their degree of malignancy. The difficulty of being sure of the thymic origin of these growths has led in recent times to a tendency to include only tumours which are removable entire surgically in published series, a policy which over-emphasises those thymic tumours which are of low malignancy; and it was to counteract this that we included those anterior mediastinal tumours encountered post mortem which could reasonably be regarded as of thymic origin. In our series of thymic tumours all shades of behaviour are represented, from simple growths through those of local malignancy and powers of infiltration to tumours of high malignancy which rapidly metastasise widely. Thus the so-called oval-celled thymoma and lympho-epithelioma have in our experience proved quite benign, slowly growing encapsuled tumours with no metastases in any of our examples. The spindle-celled tumours are a little more aggressive, but no metastases have been observed ; one of the patients in this group died from compression of the superior vena cava. The differentiated epithelial thymomas are at first enclosed in a fibrous capsule but later show signs of local invasiveness and may eventually metastasise within the chest and rarely outside it. Our example of a myasthenic with an epithelial thymoma which led to deposits in the pelvis appears to be one of the first on record which had extended outside the thorax. Granulomatous thymomas are at first circumscribed, but are rarely encountered at this stage ; they soon infiltrate surrounding structures, especially the superior vena cava, and early give rise to glandular metastases. They react very well to irradiation though they eventually prove fatal after a variable number of years. The lymphosarcomas, undifferentiated carcinomas and teratomas are uniformly highly lethal, death usually ensuing within the year. It is notable that these last three categories of ominous significance make up just half the total number of cases.

The features of the granulomatous thymoma, a tumour in which there is a granulomatous response to malignant epithelial cells and with a resemblance to Hodgkin's tissue, were clearly set out by Lowenhaupt (1948) and by Eisenberg and Sahyoun (1950). Nevertheless many writers are unconvinced by the arguments for regarding this as an epithelial tumour, considering it merely as Hodgkin's disease originating in the thymus. Not only do we accept this neoplasm as primarily epithelial in origin, but finding difficulty in applying Eisenberg and Sahyoun's criteria for differentiating these granulomatous thymomas from Hodgkin's disease, the suggestion has been made (Thomson, 1955) that Hodgkin's disease represents a metastatic dissemination of more malignant varieties of granulomatous thymomas. This matter is at present *sub judice*. In considering the merits of the theory it must be remembered how often ectopic thymic tissue is found in the neck and elsewhere, and the possibility of the neoplasm arising in such foci should be kept in mind. Furthermore, though Lowenhaupt (1948) designated this type of tumour " Carcinoma of Granulomatous Pattern (Carcinoma of Late Hassall's Corpuscles) ; Thymic Hodgkin's Disease " anything resembling a Hassall's corpuscle in these granulomatous tumours is in our experience unusual, and it may be that a similar granulomatous response is sometimes evoked by epithelial neoplasms arising in other structures of branchial origin, normal or ectopic, such as branchial cysts.

There is a further possibility of confusion between thymic tumours and " malignant reticuloses " Some types of undifferentiated epithelial thymoma have a marked similarity to reticulum cell sarcoma, that convenient designation for so many poorly differentiated neoplasms, and it is pertinent that in Roulet's (1930) original paper on reticulosarcoma the anterior mediastinum was specified as a favourite site for these neoplasms, which in some instances were actually stated to be massively replacing the thymus.

The association with myasthenia gravis forms another interesting aspect of thymic neoplasia. In a purely surgical series the frequency of tumours associated with myasthenia gravis is likely to be exaggerated, because this condition often calls for the removal of the thymus, in which tumours at an early stage of development may be found. In the present series only 8 of the 66 cases, or 12 per cent, showed this feature. In 4 of these patients the tumour was of the differentiated epithelial type, 3 had lympho-epitheliomatous thymomas and in the remaining patient the tumour was oval-celled. In no case was myasthenia gravis associated with granulomatous or undifferentiated epithelial tumours, or with lymphosarcoma or teratoma of the thymus. We have not found lymphocytic infiltration to be an invariable or striking feature of the tumours from myasthenics. Two of these 8 patients with myasthenia were operated upon during the past year and both are alive and well. Three have died as a result of myasthenia and the remaining 3 are alive and well on a reduced dosage of prostigmine up to 6½ years after thymectomy. Other endocrine abnormalities which have previously been reported in association with thymic tumours (Leyton, Turnbull and Bratton, 1931) have not been noted in this series and no cases of the type of anaemia described by Chalmers and Boheimer (1954) have been found.

Improved anaesthetic and thoracic surgical technique in recent years has resulted in the more frequent exploration of intra-thoracic tumours at a stage when their site of origin is identifiable, and has shown that thymic tumours are not as rare as was at one time supposed. The surgical pathologist is likely therefore to become increasingly familiar with the diverse histological appearances of this hitherto neglected group of neoplasms, and increasingly often faced with the necessity for identifying and forecasting the behaviour of individual examples ; it is hoped that the classification outlined above will be of assistance in this.

### SUMMARY

The histological sections of a series of 67 thymic tumours have been studied. A scheme of classification based on the histological appearances, and having some relation to the clinical behaviour of the growths, is suggested. Three main groups are recognised, epithelial, lymphoid and teratomatous ; the epithelial tumours are the most numerous and have been further subdivided into differentiated, oval- and spindle-celled, lympho-epitheliomatous, granulomatous and undifferentiated subgroups.

We gratefully acknowledge the helpful co-operation of Mr. T. Holmes Sellors, who performed most of the thymectomies on the patients in this series, in the preparation of this paper. We also wish to thank all those other members of the Consultant Staff of the Middlesex Hospital under whose care these patients were, particularly Professor B. W. Windeyer and Mr. D. H. Patey, for permission to abstract the clinical details. It is a pleasure to record our gratitude to Mr. J. W. Jackson of the Middlesex and Harefield Hospitals for his willing assistance with the surgical aspects and follow-up information on the operation cases. For the opportunity of studying the sections of tumours removed at Harefield Hospital we are indebted to Dr. E. Nassau, and to Dr. W. Blackwood for the section of the case operated on at the National Hospital for Nervous Diseases, Queen Square, London.

The expenses of this investigation were defrayed by the British Empire Cancer Campaign.

### REFERENCES

ANDRUS, W. DE W. AND FOOT, N. C.—(1937) *J. thorac. Surg.*, **6**, 648.
CASTLEMAN, B.—(1955) " Tumors of the Thymus Gland " in ' Atlas of Tumor Pathology' '. Section V, Fascicle 19. Washington (Armed Forces Institute of Pathology).
CHALMERS, J. N. M. AND BOHEIMER, K.—(1954) *Brit. med. J.*, ii, 1514.
EISENBERG, S. J. AND SAHYOUN, P. F.—(1950) *Arch. Path.*, **49**, 404.
EWING, J.—(1929) *Amer. J. Path.*, **5**, 99.
IVERSON, L.—(1956) *Ibid.*, **32**, 695.
LEYTON, O., TURNBULL, H. M. AND BRATTON, A. B.—(1931) *J. Path. Bact.*, **34**, 635.
LOWENHAUPT, E.—(1948) *Cancer*, **1**, 547.
REID, H. AND MARCUS, R.—(1949) *Brit. J. Surg.*, **36**, 271.
ROULET, F.—(1930) *Virchows Arch.*, **277**, 15.
SCHLUMBERGER, H. G.—(1951) " Tumors of the Mediastinum " in ' Atlas of Tumor Pathology'. Section V, Fascicle 18. Washington (Armed Forces Institute of Pathology).
SYMMERS, D.—(1933) *Ann. Surg.*, **95**, 544.
THOMSON, A. D.—(1955) *Brit. J. Cancer*. **9**, 37.

### EXPLANATION OF PLATES

FIG. 1.—Low power view showing the clear distinction between the paler medullary region of the thymus, in which Hassall's corpuscles can be seen, and the densely lymphocytic cortex. × 40.

FIG. 2.—Row of basal epithelial cells at the periphery of the cortex of the thymus. The epithelial cells derived from them are a little larger and paler, but not always easily distinguishable from the large numbers of lymphocytes in the cortex. × 280.

FIG. 3.—Medullary region of the hyperplastic, but otherwise normal thymus from a case of myasthenia shown in the previous figures. A syncytial group of epithelial cell nuclei (right centre) and part of a Hassall's corpuscle on the left. × 280.

FIG. 4.—Sheets of epithelial cells from a thymic tumour. There is degeneration of the stroma in which a small blood vessel lies at the centre. × 150.

FIG. 5.—Another field from the tumour shown in Fig. 4. The epithelial cells are swollen and pale, and there are numerous foam cells and cholesterol crystal clefts present. × 115.

FIG. 6.—Part of another epithelial tumour of the thymus similar to that shown in Fig. 3 and 4, except that there is patchy but heavy lymphocytic infiltration, as seen in the top right corner. × 295.

FIG. 7.—High power view of an epithelial thymic tumour, the cells of which are uniform in size and staining, and which, in this field, vary in shape from squamous to oval. × 250.

FIG. 8.—High power view of an epithelial thymic tumour, the cells of which are approximately oval in outline. × 280.

FIG. 9.—High power view of an epithelial thymic tumour composed of spindle-shaped cells distinguishable with difficulty from the young fibrocytes of the stroma at bottom right. × 280.

FIG. 10.—Another tumour similar to that in Fig. 9 showing the change in the general appearance of the growth caused by heavy infiltration with lymphocytes. ×280.

FIG. 11.—High power view of the lympho-epitheliomatous type. The uniform large pale epithelial cells are regularly interspersed with and obscured by lymphocytes. × 280.

FIG. 12.—A poorly differentiated epithelial tumour, the cells vary in size and staining and mitotic figures are numerous. × 280.

FIG. 13.—Part of a granulomatous thymoma in which the epithelial cells are forming a coherent sheet. × 295.

FIG. 14.—Very high power field from a granulomatous thymoma showing an epithelial cell in mitosis, a mirror image giant cell and some eosinophils. × 570.

FIG. 15.—An area from the tumour shown in Fig. 13 in which early fibrosis is apparent. × 295.

FIG. 16.—A granulomatous thymoma showing epithelial cells in an area of considerable fibrosis. × 295.

FIG. 17.—Extensive fibrosis in a granulomatous thymoma. × 280.

FIG. 18.—An anaplastic carcinoma of the thymus showing fibrous stroma to the right, a band of tumour cells in which mitoses are evident, and part of a large area of necrosis on the left. × 280.

FIG. 19.—Nodule of normal thymic tissue, a chance encounter in a normally situated thyroid. × 9.

FIG. 20.—Two Hassall's corpuscles from the hyperplastic thymus of a case of myasthenia gravis. The upper corpuscle is calcified and has a foreign body giant cell applied to it; the lower small corpuscle has a few central keratinised cells and a cell clearly showing keratohyaline granules. × 295.

FIG. 21.—An epithelial thymic tumour showing the characteristic trabeculae of fibrous tissue. One lobule of the tumour which is heavily infiltrated with lymphocytes is conspicuously darker than the pale purely epithelial areas. × 35.

FIG. 22.—Hassall's corpuscle, showing keratinisation, from a lymph node metastasis of a differentiated epithelial thymic tumour. × 295.

FIG. 23.—Invasion of myocardium by an undifferentiated epithelial thymic tumour. × 120.

FIG. 24.—Very low power view of an epithelial thymic tumour showing broad bands of fibrous tissue; tumour nodules infiltrated with lymphocytes appear darker. ×15.

FIG. 25.—Two Hassall's corpuscles from a metastasis of a differentiated epithelial thymic tumour, with lymphocytic infiltration around. × 295.

FIG. 26.—From an abdominal metastasis of an epidermoid thymic tumour. Early cystic degeneration of the stroma at the centre with the basal layer of epithelium at its periphery giving a semblance of tubule formation. × 115.

FIG. 27.—Low power view of an oval-celled thymic tumour; the same tumour as shown in Fig. 8. × 85.

FIG. 28.—Low power view showing the general arrangement of the spindle celled thymic tumour illustrated in Fig. 9. × 85.

FIG. 29.—Low power view of the tumour shown in Fig. 7, to show numerous cystic spaces. × 85.

FIG. 30 and 31.—An owl-like cell and a multinucleate cell from a thymic tumour of the granulomatous variety. × 280.

FIG. 32.—Multinucleate epithelial syncytium from a normal thymus to compare with Fig. 31. × 280.

FIG. 33.—Lymphosarcoma of thymus. × 295.

FIG. 34.—Same tumour as previous figure, showing an area of necrosis with calcified Hassall's corpuscles. × 295.

FIG. 35.—Cartilage and epithelial tubules from a teratomatous tumour at the site of the thymus. × 75.

FIG. 36.—Squamous and columnar epithelium from the same tumour as shown in the previous figure. × 75.